# Three-dimensional wall-thickness distributions of unruptured intracranial aneurysms characterized by micro-computed tomography

**DOI:** 10.1007/s10237-024-01835-5

**Published:** 2024-03-15

**Authors:** Yasutaka Tobe, Takanobu Yagi, Koichi Kawamura, Kenta Suto, Yoichi Sawada, Yoshifumi Hayashi, Hirotaka Yoshida, Kazutoshi Nishitani, Yoshifumi Okada, Shigemi Kitahara, Mitsuo Umezu

**Affiliations:** 1 Center for Advanced Biomedical Sciences, Waseda University, 2-2 Wakamatsucho Shinjukuku, Tokyo, 162-8480 Japan; 2 Second Department of Pathology, Akita University, Akita, Japan; 3 Department of Health and Welfare Science, Okayama Prefectural University, Okayama, Japan; 4 Department of Neurosurgery, Kitahara International Hospital, Tokyo, Japan; 5 Department of Neurosurgery, Tokyo General Hospital, Tokyo, Japan

**Keywords:** Intracranial aneurysm, Wall thickness, Blood pressure, Wall stress, Remodeling

## Abstract

Aneurysmal rupture is associated with wall thinning, but the mechanism is poorly understood. This study aimed to characterize the three-dimensional wall-thickness distributions of unruptured intracranial aneurysms. Five aneurysmal tissues were investigated using micro-computed tomography. First, the wall thickness was related to the aneurysmal wall appearances during surgery. The median wall thicknesses of the translucent and non-translucent walls were 50.56 and 155.93 µm, respectively (*p* < 0.05) with significant variation in the non-translucent wall thicknesses (*p* < 0.05). The three-dimensional observations characterized the spatial variation of wall thicknesses. Thin walls showed a uniform thickness profile ranging from 10 to 40 µm, whereas thick walls presented a peaked thickness profile ranging from 300 to 500 µm. In transition walls, the profile undulated due to the formation of focal thin/thick spots. Overall, the aneurysmal wall thicknesses were strongly site-dependent and spatially varied by 10 to 40 times within individual cases. Aneurysmal walls are exposed to wall stress driven by blood pressure. In theory, the magnitude of wall stress is inversely proportional to wall thickness. Thus, the observed spatial variation of wall thickness may increase the spatial variation of wall stress to a similar extent. The irregular wall thickness may yield stress concentration. The observed thin walls and focal thin spots may be caused by excessive wall stresses at the range of mechanical failure inducing wall injuries, such as microscopic tears, during aneurysmal enlargement. The present results suggested that blood pressure (wall stress) may have a potential of acting as a trigger of aneurysmal wall injury.

## Introduction

The prevalence of unruptured intracranial aneurysms (UIAs) reaches 3–6% in adults (Rinkel et al. 1998; Vlak et al. 2011). UIA rupture is the most frequent cause of subarachnoid hemorrhage, occurring in approximately 1% of UIAs per year (Wiebers et al. 2003; Morita et al. 2012; Greving et al. 2014). Given the high mortality rate (30–50%) of UIA rupture (Brisman et al. 2006; Nieuwkamp et al. 2009), identifying high-risk UIAs is an important task. The rupture risk of UIAs is associated with various morphological factors—aneurysmal size, location, and shape—showing distinct profiles between unruptured and ruptured aneurysms (Wiebers et al. 2003; Morita et al. 2012; Greving et al. 2014).

Aneurysmal wall appearances are associated with aneurysmal size. Mizoi et al. (1996) found entirely translucent walls in small aneurysms and partially or entirely non-translucent walls in large aneurysms. Their observations were later consolidated by Kadasi et al. (2013) and Song et al. (2015). These studies analyzed the color maps of intraoperative videos and classified aneurysmal walls as translucent (TL) or non-translucent (NTL). The actual thicknesses of TL and NTL walls are not yet characterized. These studies also suggest that aneurysmal wall remodeling may contain two counter pathways of wall thinning and thickening.

Aneurysmal ruptures are associated with wall thinning. Frösen et al. (2004) and Tulamo et al. (2010) demonstrated different wall structures in unruptured and ruptured aneurysmal walls. They observed extremely thin and decellularized wall structures with an organized luminal thrombus only in ruptured aneurysms (Tulamo et al. 2010). Aneurysmal wall remodeling seems to be influenced by aneurysmal hemodynamics. Suzuki et al. (2016) and Cebral et al. (2019) showed that aneurysmal walls exposed to impinging flows are translucent. This knowledge suggests an association of wall thinning with flow impingement.

A modern theory of aneurysmal wall pathology posits that aneurysmal wall remodeling is mediated by flow-induced inflammation; specifically, high wall shear stress triggers endothelial dysfunction, de-endothelialization, and macrophage infiltration, inducing the formation of luminal thrombi followed by mural cell death and matrix degradation (Frösen et al. 2012, 2019; Cebral et al. 2017). However, our pathological understanding of aneurysmal hemodynamics remains poor. To our knowledge, only one study has analyzed the aneurysmal hemodynamics with aneurysmal wall pathology (Cebral et al. 2017). The authors independently characterized and statistically linked the hemodynamics and pathology. Thus, none of the previous studies has combined hemodynamics with pathology from a phenomenological perspective.

Aneurysmal walls are exposed to two types of mechanical stresses: wall shear stress and wall stress. Wall shear stress is driven by blood viscosity and wall stress by blood pressure. The magnitude of these stresses can be estimated with a theory of the Hagen–Poiseuille flow and thin-walled pressure vessels, respectively, as follows:

1 $$\tau_{w} = \frac{4\mu Q}{{\pi R^{3} }}$$

2 $$\sigma_{\theta } = \frac{{{\text{PR}}}}{T}$$

where ${\tau }_{w}$, ${\sigma }_{\theta }$, and $T$ are the wall shear stress, tangential wall stress, and wall thickness of the vessel, respectively, $\mu$ is the blood viscosity, $Q$ is the flow rate, $R$ is the radius, and $P$ is the trans-wall pressure difference. Taking representative values ($\mu$ = 0.004 Pas, $Q$ = 250 mL/min, $R$ = 2.5 mm, $P$ = 100 mmHg, and $T$ = 0.5 mm, assuming the internal carotid artery), ${\tau }_{w}$ is 1.4 Pa, and ${\sigma }_{\theta }$ is as high as 66,500 Pa. Despite this large difference (10^4^ orders), research has focused solely on the wall shear stress over the past three decades. The possible involvement of wall stress has been neglected due to a widely accepted assumption that endothelial cell dysfunction is a trigger of aneurysmal formation and progression (Frösen et al. 2019). Undoubtedly, endothelial cells can respond to wall shear stress (e.g., Zhou et al. 2014), but smooth muscle cells (SMCs) can respond to wall stress (e.g., Lacolley et al. 2017). Aneurysmal formation has historically been related to medial defects, including loss of internal elastic lamina (e.g., Forbus 1930). Besides, aneurysmal rupture is related to death of mural cells such as SMCs (Frösen 2014). In vitro studies have suggested that SMC apoptosis can be induced by altered mechanical stretches (Mantella et al. 2015). SMC apoptosis has also been observed in human intracranial aneurysms, especially in ruptured aneurysms (Frösen et al. 2012).

Aforementioned studies motivated us to study the role of wall stress in aneurysmal wall pathology. The lack of measurement techniques, however, prevents researchers from gaining full understanding of the effect of wall stress, but as shown by Eq. (2), the wall stress is inversely proportional to wall thickness. To understand the role of wall stress in aneurysmal wall remodeling, we present the first characterization of three-dimensional wall-thickness distributions of UIAs using micro-computed tomography. Over the past decade, our bioengineering laboratory in conjunction with medical institutions has linked aneurysmal wall mechanics to aneurysmal wall pathology using human samples harvested during craniotomy. To examine the wall thicknesses of these samples, we dehydrated the samples and subjected them to micro-computed tomography, which were followed by luminal surface studies using scanning electron microscopy and ultrastructural wall studies using transmission electron microscopy. This first report characterizes the three-dimensional wall-thickness distributions of UIAs. We first assessed the shrinkage rate during tissue dehydration using cadaver-derived intracranial arteries. We then related the aneurysmal wall thickness to aneurysmal wall appearances, as done in the previous reports (Mizoi et al. 1996; Kadasi et al. 2013; Song et al. 2015). Finally, the three-dimensional walls were characterized in terms of their thicknesses.

## Materials and methods

### Samples

**Fig. 1:**
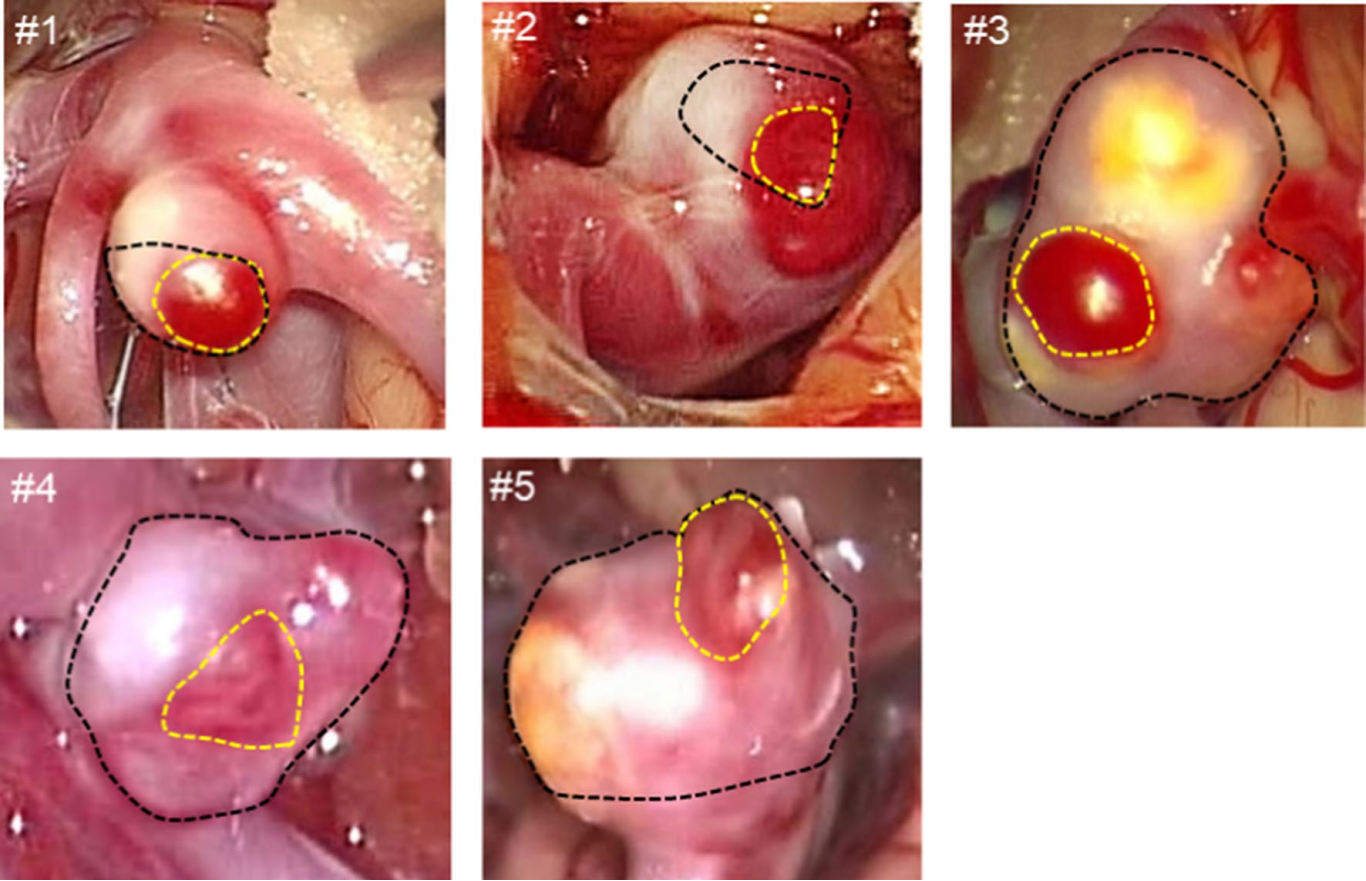
Intraoperative appearances of the intracranial aneurysms. Areas enclosed by the black dotted lines were observed in intraoperative videos of the harvested tissues. Within the observable area, the wall types were classified as translucent (TL) or non-translucent (NTL). The yellow dotted lines delineate the TL walls. The outsides of the TL walls are classified as NTL walls

Investigation of the aneurysmal samples was approved by the Ethics Committee of Kitahara International Hospital, Japan. All patients gave informed consent to use of their clinical data. Investigation of the harvested samples was approved by the Ethics Committee of Waseda University, Japan. All methods accorded with relevant guidelines and regulations. Craniotomy clippings of UIAs were made in 44 patients from April of 2011 to June of 2012, and nine aneurysmal tissues were harvested. Five of the harvested tissues included TL and NTL walls; the remaining four presented NTL walls only. The former five tissues were investigated in the present study. Table 1 summarizes the patient backgrounds. The patients were aged 67.2 ± 6.1 y (mean ± SD; mean = arithmetic average, SD = standard deviation). All aneurysms were located in the middle cerebral artery (MCA) and were sized 4.8 ± 1.0 mm (mean ± SD). The wall types (TL or NTL) were classified from the color maps of intraoperative videos. TL walls were unanimously assessed as red by three independent observers (Fig. 1). Ambiguous sites that failed to gain unanimous agreement, such as naturally reddish walls or walls rendered reddish/red by surgical contacts, were classified as NTL walls.

**Table 1:** Background details of patients with intracranial aneurysms

Case no.	Age	Gender	AN Loc	AN size mm
1	69	M	MCA	4
2	68	F	MCA	5.8
3	76	F	MCA	5.4
4	63	F	MCA	3.4
5	60	M	MCA	5.4

### Wall-thickness measurements

**Fig. 2:**
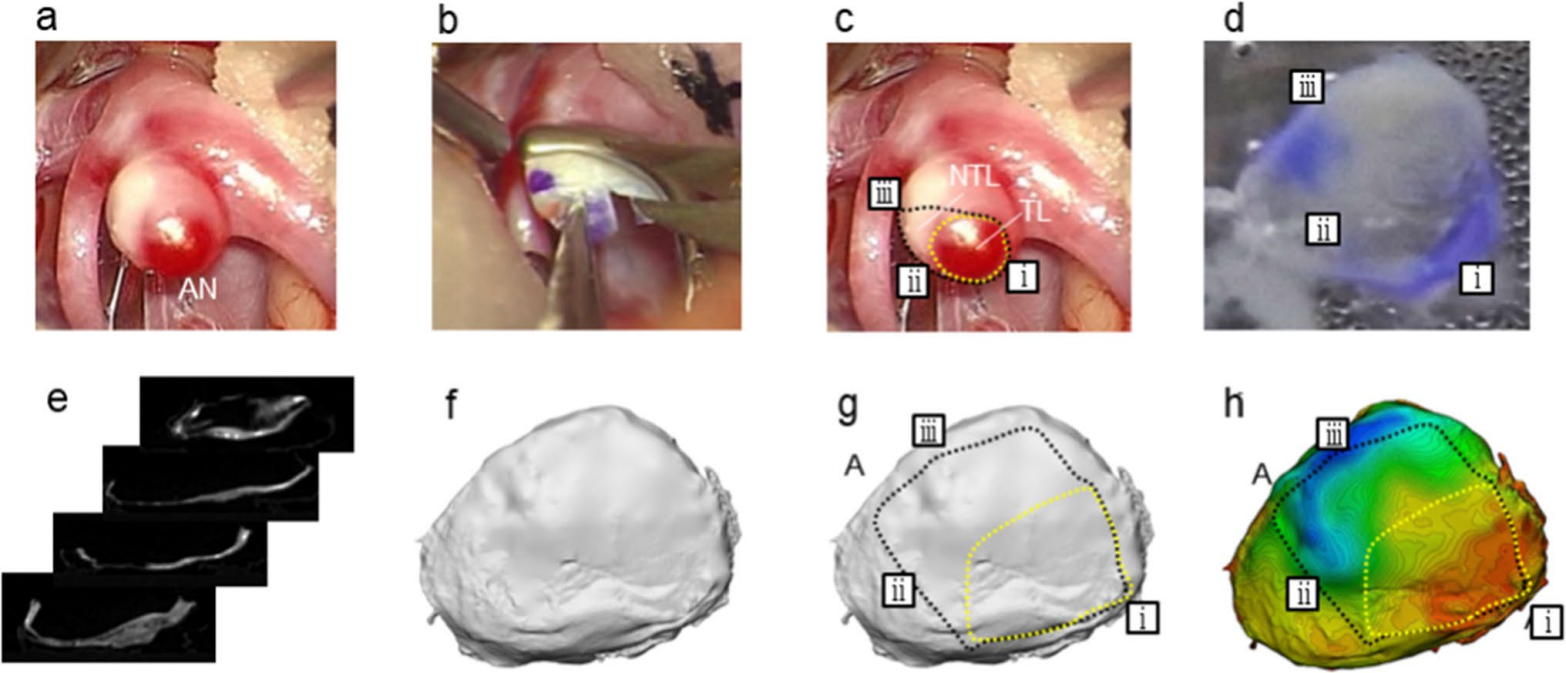
Experimental procedure for measuring three-dimensional aneurysmal wall thicknesses: **a** intraoperative appearance of an aneurysm (AN), **b** marking and harvesting, **c** wall-type classification, **d** harvesting of tissue, **e** tissue dehydration and micro-computed tomography measurement, **f** three-dimensional volume reconstruction, de-noising, and remeshing, **g** wall-type mapping and segmentation, and **h** three-dimensional wall-thickness distribution. The black and yellow dotted lines are explained in the caption of Fig. 1. The orientation of the three-dimensional model matches that of the intraoperative appearance before marking and harvesting

Figure 2 outlines the procedures for measuring thickness from micro-computed tomography images. Aneurysms exposed in surrounding tissues were marked with surgical ink. After placing a clip at a neck of aneurysm, an aneurysmal dome was harvested. The locations and orientations of the harvested tissues were indicated by the ink markers. The harvested tissues were gently washed with phosphate-buffered saline, fixed in 2% glutaraldehyde solution for 24 h at 4 °C, post-fixed in 4% osmium acid solution for 2 h at 4 °C, dehydrated in an ethanol series (30–100%), immersed in tertiary-butyl alcohol and ethanol solutions with different volume ratios (3:7, 1:1, and 1:0), freeze-dried under a vacuum for 2 h (VFD-21S; Vacuum Device Corp., Japan), and coated with platinum–palladium by a magnetron sputter coater (MSP-10; Vacuum Device Corp., Japan). The dehydrated tissues were scanned using micro-computed tomography (TDM1300-IS; Yamato Kagaku, Japan). The spatial resolution was 5.4–14.6 µm depending on the tissue size. A series of images was exported to volume-rendering software (Mimics; Materialise, Belgium). The resulting stereolithography (STL) models were imported to computer-aided design software (3-Matic; Materialize, Belgium). After careful de-noising, the STL models were re-triangulated at a spatial resolution of 10 µm. Wall-type mapping and segmentation were carried out in the STL model. To measure the wall thickness, a mid-plane between the inner and outer surfaces was generated by the software. The distance to these surfaces in the direction perpendicular to the middle plane was then defined as the wall thickness. The mean wall thickness was computed as the areal mean by considering the area of triangle meshes, which was used to extract the standard deviations of wall thickness.

### Statistical analysis

The descriptive statistics (mean, SD, 95% confidence interval, median, maximum, and minimum) were computed. The median thicknesses of the TL and NTL walls in individual cases were compared using the two-tailed Mann–Whitney U-test, and the medians and standard deviations in the five cases were evaluated using the two-tailed Wilcoxon test. All statistical analyses were performed using IBM Statistical Package for the Social Sciences (SPSS) ver. 25 (IBM Corp., Tokyo, Japan). The significance level (*α*) of all tests was set at 0.05, and the *p*-values were adjusted as necessary to account for repeated testing.

## Results

### Effect of tissue shrinkage due to dehydration

**Fig. 3:**
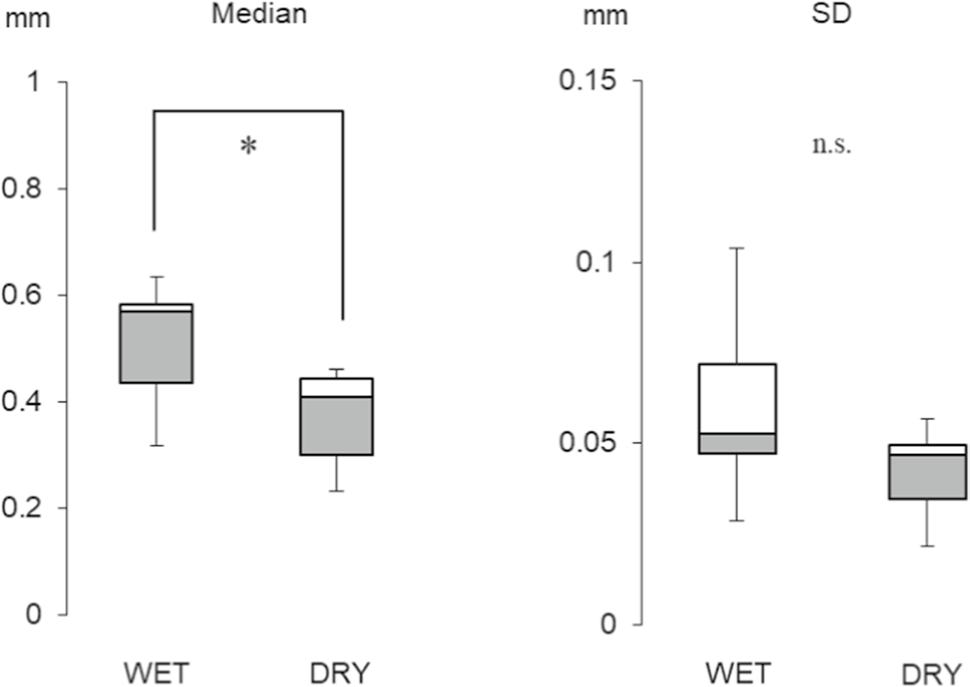
Effect of tissue dehydration on wall-thickness measurements of intracranial arteries. The medians and standard deviations of the WET and DRY were calculated from those of six cases (Table 2) (*: *p* < 0.05)

We first evaluated the effect of tissue shrinkage caused by dehydration using the intracranial arteries from cadavers. These samples were denoted and stored in formalin at Akita University, Japan, for research and educational purposes with informed consent and utilized under relevant guidelines and regulations. Experiments were performed on six arteries from six cadavers (four males and two females, age 62 ± 14 y [mean ± SD]: three in the middle cerebral arteries and three in the internal carotid arteries). The wall thicknesses of the WET (originally prepared) and DRY (dehydrated) samples were measured at six randomly selected sampling points. The locations of the sampling points were matched in the WET and DRY samples. The median thickness in each case and the medians and SDs among the six cases were analyzed using a two-tailed Wilcoxon test as described above. On the per-case basis, the median thicknesses did not significantly differ between the WET and DRY samples (Table 2), but the six-case median thickness of the WET samples (0.57 mm) differed from that of the DRY samples (0.41 mm) (see Fig. 3). Thus, the tissue shrinkage rate due to dehydration was assumed as 30%. For clarity, the following results do not account for shrinkage.

**Table 2:** Effect of tissue dehydration on wall-thickness measurements of intracranial arteries (Unit = mm)

Case no.		*N*	Mean ± SD	95%CI low–high	Median	Min.–Max.	Two-tailed Wilcoxon Z	Adjusted *p*-value
1	WET	6	0.33 ± 0.05	0.28–0.38	0.32	0.28–0.40	2.20	*p* = 0.168: n.s
	DRY	6	0.24 ± 0.02	0.22–0.26	0.23	0.22–0.28		
2	WET	6	0.59 ± 0.05	0.53–0.64	0.58	0.52–0.66	2.20	*p* = 0.168: n.s
	DRY	6	0.42 ± 0.05	0.38–0.48	0.42	0.38–0.52		
3	WET	6	0.53 ± 0.10	0.42–0.64	0.56	0.35–0.63	2.20	*p* = 0.168: n.s
	DRY	6	0.43 ± 0.06	0.37–0.49	0.46	0.33–0.47		
4	WET	6	0.62 ± 0.06	0.56–0.68	0.63	0.54–0.68	2.20	*p* = 0.168: n.s
	DRY	6	0.47 ± 0.04	0.42–0.51	0.45	0.43–0.54		
5	WET	6	0.60 ± 0.08	0.52–0.68	0.58	0.52–0.73	2.20	*p* = 0.168: n.s
	DRY	6	0.41 ± 0.05	0.36–0.46	0.40	0.37–0.49		
6	WET	6	0.39 ± 0.03	0.36–0.41	0.39	0.34–0.42	2.20	*p* = 0.168: n.s
	DRY	6	0.25 ± 0.03	0.23–0.28	0.27	0.19–0.27		

WET and DRY refer to non-dehydrated and dehydrated tissue samples, respectively. The sampling points *N* were matched in WET and DRY tissues; Mean: arithmetic average, *SD* standard deviation, *CI* confidence interval, *Min* minimum, and *Max* maximum. Statistical analyses were applied to the medians of the WET and DRY samples. Adjusted *p*-value: The calculated p-value was multiplied by the number of statistical significance tests performed

### Comparison of translucent and non-translucent wall thickness

**Fig. 4:**
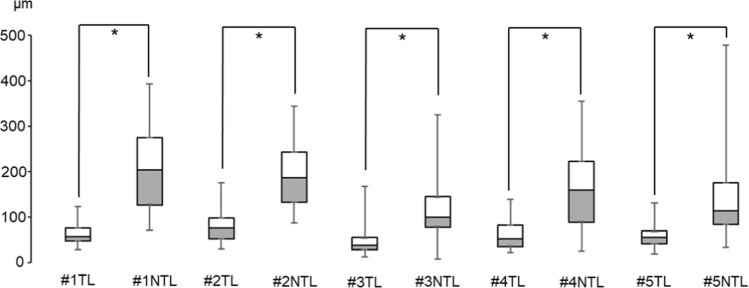
Comparison of TL and NTL aneurysmal wall thicknesses in each case. Statistical analyses were applied to the median wall thicknesses, and the calculated p-values were multiplied by the number of statistical significance tests performed (adjusted *p*-value) (*: *p* < 0.001)

**Fig. 5:**
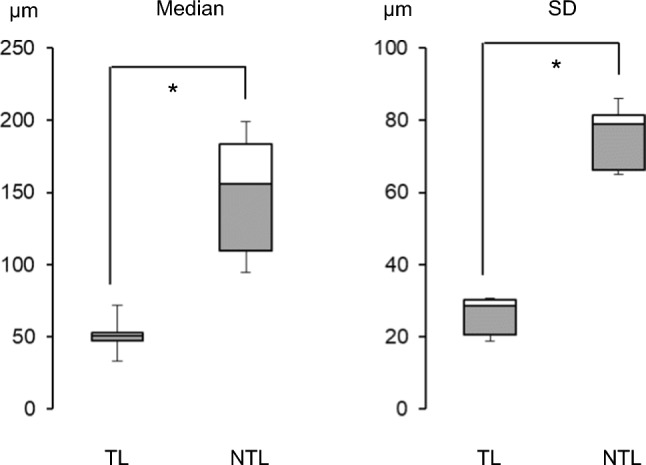
Comparison of TL and NTL aneurysmal wall thicknesses in the five cases. The medians and standard deviations were calculated from those of the five cases (Table 3) (*: *p* < 0.05)

The TL and NTL wall thicknesses were compared in each case (Table 3 and Fig. 4) and among the five cases (Fig. 5). In each case (taking median values), the TL wall was significantly thinner than its NTL counterpart. The NTL wall thicknesses were more variable than the TL walls. The minimum wall thickness of the NTL wall overlapped with the range of TL wall thicknesses. In particular, the minimum thicknesses of the TL and NTL walls in samples #3–5 were similar. These data demonstrated that the NTL wall included thin walls similar to the TL wall. The five-case comparison of the TL and NTL wall thicknesses (Fig. 5) showed significant differences between both the medians and standard deviations, revealing that the TL walls were both thinner and more uniform than the NTL walls.

**Table 3:** Comparison of translucent (TL) and non-translucent (NTL) aneurysmal wall thicknesses

Case no.		*N*	Mean ± SD (µm)	95%CI low–high	Median (µm)	Min.–Max. (µm)	Two-tailed Mann–Whitney U	Adjusted *p*-value
1	TL	9169	59.14 ± 20.70	58.72–59.56	53.08	23.86–118.32	7,747,371.50	*p* < 0.001
	NTL	55,571	204.56 ± 86.10	203.84–205.27	199.32	67.09–391.14		
2	TL	16,940	74.30 ± 30.15	73.85–74.76	71.85	26.09–171.99	12,558,539.50	*p* < 0.001
	NTL	16,627	188.05 ± 66.13	187.06–189.05	183.80	84.44–342.56		
3	TL	30,967	44.10 ± 30.74	43.76–44.44	32.83	8.53–163.96	224,670,087.00	*p* < 0.001
	NTL	70,788	117.83 ± 65.08	117.35–118.30	94.89	2.91–322.11		
4	TL	10,944	55.57 ± 28.52	55.03–56.10	47.19	17.64–135.41	92,106,718.00	*p* < 0.001
	NTL	70,544	158.33 ± 81.55	157.73–158.93	155.93	21.43–352.61		
5	TL	22,645	52.20 ± 18.69	51.96–52.45	50.56	13.88–127.04	207,848,738.50	*p* < 0.001
	NTL	110,938	135.41 ± 78.88	134.94–135.87	109.39	28.46–476.92		

*N* denotes the number of sampling points. Statistical analyses were applied to the median TL and NTL wall thicknesses in each case. Adjusted *p*-value: The calculated p-value was multiplied by the number of statistical significance tests performed

### Three-dimensional wall-thickness distribution

**Fig. 6:**
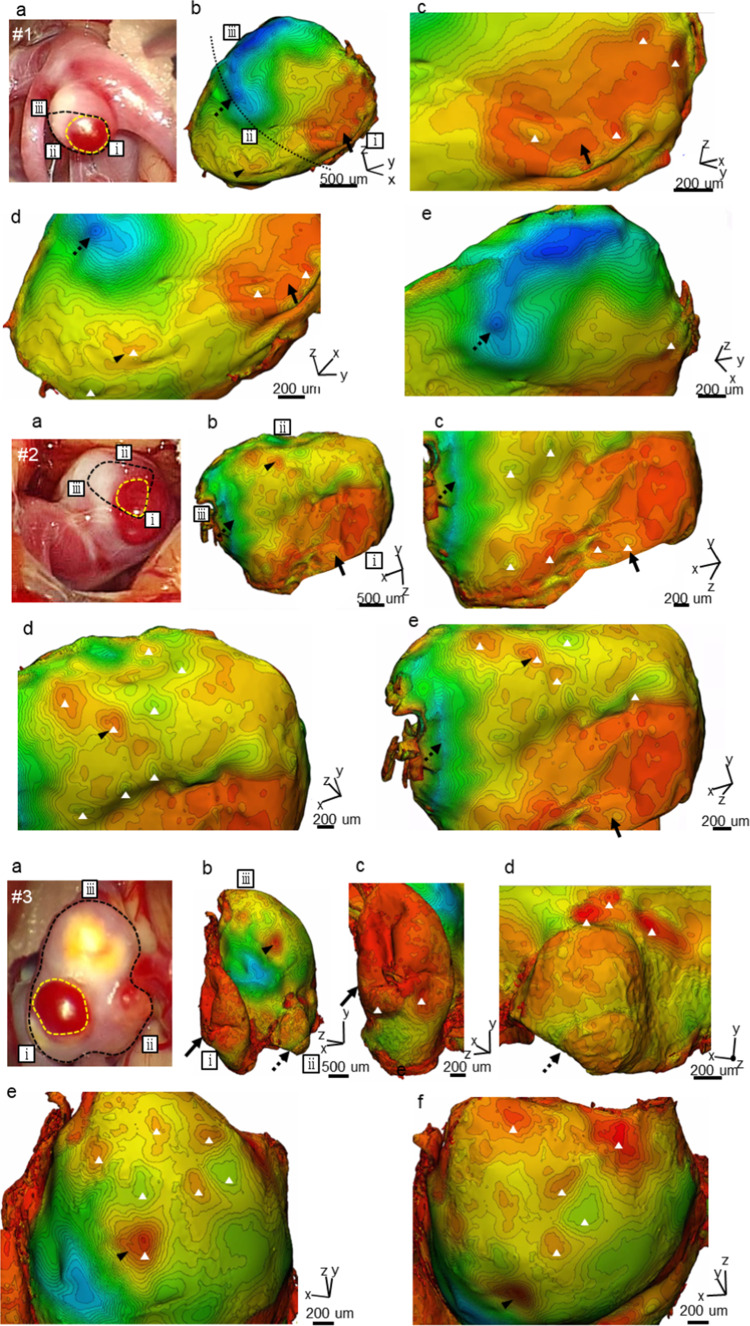

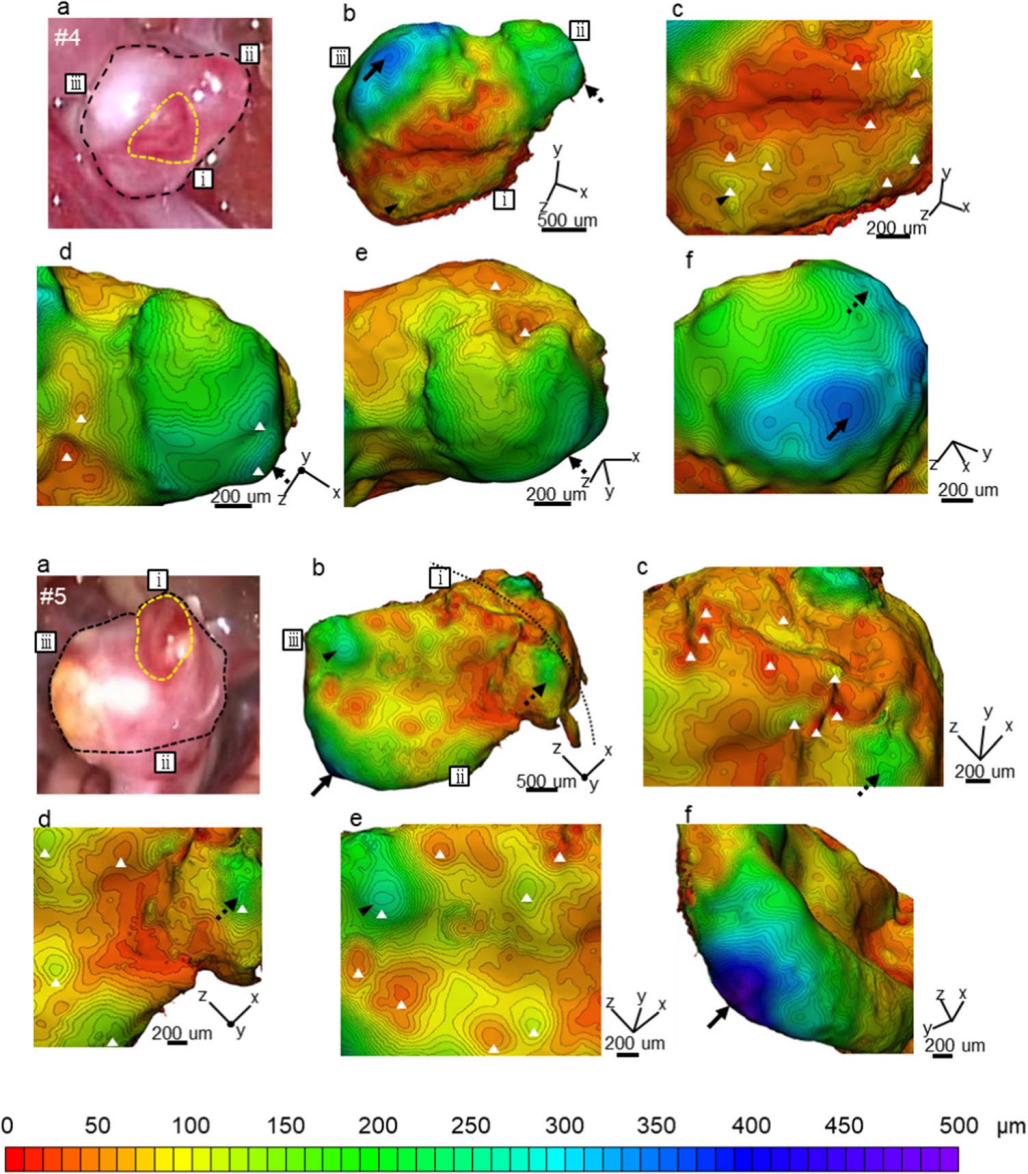
Three-dimensional wall-thickness distributions of five intracranial aneurysms (#1,2,3,4,5): **a** intraoperative appearances delineating the observable areas in the harvested tissues (black dotted lines) and the locations of TL walls (yellow dotted lines); **b** overall three-dimensional wall-thickness distribution in the same orientation as **a**; and **c**–**f** zoomed-in views in different orientations. Black arrows and arrowheads point to the same locations. White triangles indicate the locations of focal thin/thick spots. The fine dotted lines in #1b and #5b indicate the borders of the observable regions in the intraoperative video

Figure 6 summarizes the three-dimensional wall-thickness distributions of UIAs by micro-computed tomography. Panels (a) of this figure show the intraoperative appearances of the harvested tissues with observable aneurysms. The locations of the TL walls are also indicated. Panels (b) show the overall three-dimensional wall-thickness distribution along the orientation of (a), along with zoomed-in views (panels (c–f)).

The median TL wall thickness in case #1 was approximately 50 µm (Table 3). The TL wall included a region of uniform 40-μm thickness profile evidenced by low-density contour lines (Fig. 6, #1c). Surrounding the uniform profile is a formation of focal thin/thick spots with thicknesses ranging from 30 to 70 µm, as evidenced by focally enclosed contour lines (#1c). In the further surrounding region, persistent focal thin/thick spots with thicknesses ranging from 70 to 120 µm were observed (#1d, e). The thickness sharply increased at the border of the TL and NTL walls, as indicated by the high-density contour lines in Fig. 6 (#1d, e). The NTL wall thickness exhibited a 390-µm-thick peaked profile. Overall, the minimum and maximum wall thicknesses differed by approximately 20 times (Table 3).

The median TL wall thickness in case #2 was approximately 70 µm (Table 3). The TL wall included a region of uniform 30-µm thickness profile (Fig. 6, #2c) surrounded by a formation of focal thin/thick spots with thicknesses ranging from 30 to 120 µm (#2c). Focal thin/thick spots with thicknesses ranging from 50 to 140 µm persisted in the further surrounding region (#2d, e). The NTL wall thickness presented a 340-µm-thick peaked profile. Overall, the minimum and maximum wall thicknesses differed by approximately 10 times (Table 3).

The median TL wall thickness in case #3 was approximately 30 µm (Table 3). The TL wall included a region of uniform 20**-**µm thickness profile (Fig. 6, #3c) surrounded by a formation of focal thin spots with a thickness of 20 µm (#3c). Focal thin/thick spots with thicknesses ranging from 10 to 130 µm persisted in the further surrounding region (#3d, e, f). The NTL wall thickness included a 320-µm-thick peaked profile. Overall, the minimum and maximum wall thickness differed by approximately 40 times (Table 3).

The median TL wall thickness in case #4 was approximately 50 µm (Table 3). The TL wall included a region of a uniform 20**-**µm thickness profile (Fig. 6, #4c) surrounded by a formation of focal thin/thick spots with thicknesses ranging from 20 to 100 µm (#4c). Focal thin/thick spots with thicknesses ranging from 30 to 270 µm persisted in the further surrounding region (#4d, e). The NTL wall thickness included a 350-µm-thick peaked profile (#4f). Overall, the minimum and maximum wall thickness differed by approximately 20 times (Table 3).

The median TL wall thickness in case #5 was approximately 50 µm (Table 3). Unlike cases #1–4, the TL wall consisted of focal thin/thick spots with thicknesses ranging from 10 to 130 µm (Fig. 6, #5c), and a uniform 30-µm thickness profile appeared in the vicinity (#5d). In the surrounding region of the uniform profile, focal thin/thick spots with thicknesses ranging from 40 to 180 µm were observed (#5d). Focal thin/thick spots with thicknesses ranging from 40 to 130 µm persisted in the further surrounding region. The NTL wall thickness included a 480-µm-thick peaked profile. Overall, the minimum and maximum wall thickness differed by approximately 30 times (Table 3).

## Discussion

We first review the wall thickness of intracranial aneurysms in ex vivo measurements. In vivo measurements using magnetic resonance imaging are excluded due to their limited spatial resolution. The ex vivo aneurysmal wall thicknesses were variously measured. Macdonald et al. (2000) and Robertson et al. (2015) reported the wall thicknesses of 16–212 µm and 130–450 µm, respectively. These variations corresponded to those found among different patients. Suzuki and Ohara (1978) reported wall-thickness variations at the neck and dome within individual cases, which overall ranged from 20 to 550 µm. This wall thickness was measured as a length between the inside and outside of aneurysmal walls in pathological sections. Similar methods were adopted by Acosta et al. (2021). These wall thicknesses were potentially overestimated depending on sectioning angles and gave us only one-dimensional profile. Overall, our present results fall within similar ranges as the previous data. In the surrounding studies, Niemann et al. (2020, 2021, 2023) reported a method of reconstructing three-dimensional wall thicknesses from sections, but the three-dimensional wall-thickness distributions are not yet characterized. Gade et al. (2019) measured three-dimensional wall thicknesses from micro-computed tomography data, but their interest was given to find a link to wall calcification, not spatial variations of wall thicknesses within individual cases.

**Fig. 7:**
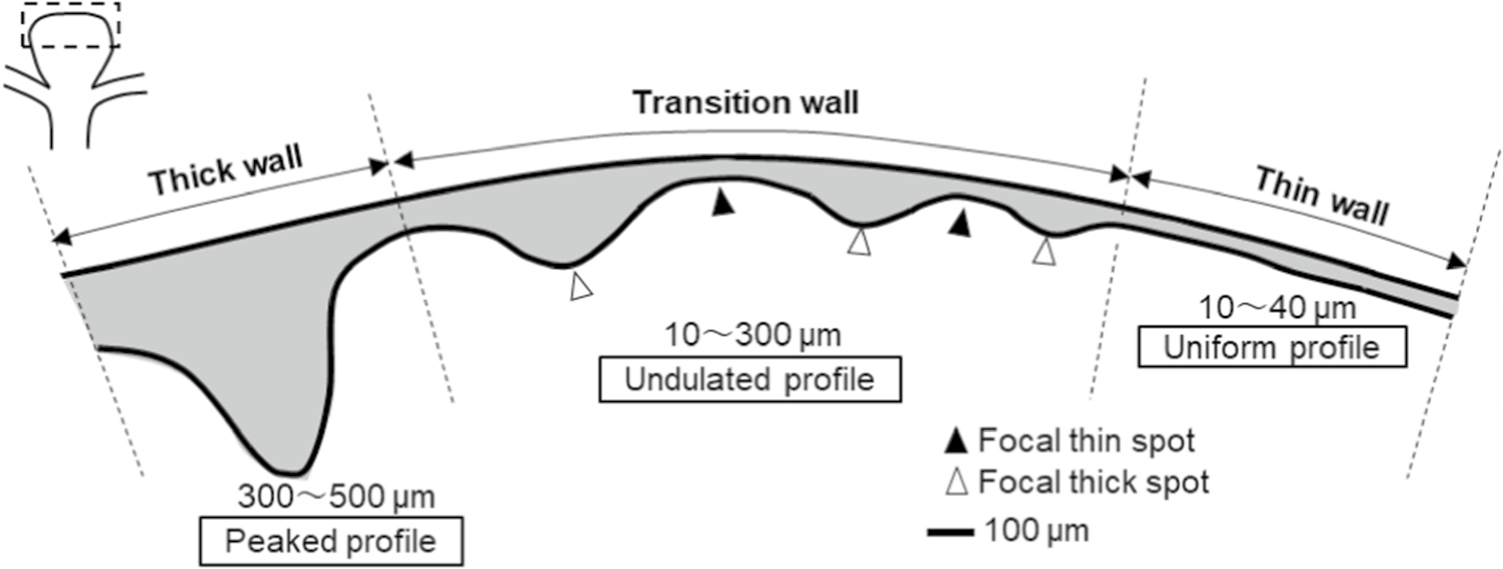
Characterization of aneurysmal wall thicknesses in a dome (ignoring the shrinkage due to tissue dehydration)

The literature review revealed no attempt to characterize the three-dimensional wall-thickness distribution of intracranial aneurysms within individual cases. To this end, the present study first related aneurysmal appearance to wall thickness. The TL walls were found to be thinner than the NTL walls in a quantitative fashion. The median TL wall thickness was approximately 50 µm, one-third that of the NTL walls. Interestingly, the TL wall exhibited a more uniform thickness profile, while the NTL wall gave significant variations including sites of thickness similar to that of the TL wall. Eventually, the three-dimensional wall-thickness distributions revealed three characteristic types of aneurysmal walls: (1) extremely thin walls with uniform thickness profiles (10–40 µm) (thin walls), (2) undulating thickness profiles due to the formation of focal thin/thick spots (10–300 µm) (transition walls), and (3) extremely thick walls with peaked thickness profiles (300–500 µm) (thick walls). These three wall types are shown in Fig. 7.

The spatial variation of the aneurysmal wall thickness is expected to reflect the coexistence of counter pathways, or wall thinning and thickening (see also Introduction), in individual aneurysms during their enlargement. Frösen et al. (2004) and Tulamo et al. (2010) demonstrated different wall structures in unruptured and ruptured aneurysmal walls. They observed extremely thin and decellularized wall structures with an organized luminal thrombus only in ruptured aneurysms (Tulamo et al. 2010). This knowledge is a core of modern aneurysmal wall pathology, which supposes that luminal thrombus acts as a promoting factor of wall injury by inducing mural cell death and matrix degradation through oxidative stresses released by inflammatory cells in luminal thrombi (Frösen et al. 2012). The present study found entirely thin walls in unruptured aneurysms, which possibly contradict the findings of Frösen et al. (2004) and Tulamo et al. (2010). Future studies should consider the spatial variation of wall structures, including wall thickness, and investigate whether thin walls are covered by luminal thrombus.

Aneurysms rupture when the wall stress surpasses the wall strength. Robertson et al. (2015) reported the ultimate stress of intracranial aneurysms using uniaxial loading tests, which ranged from 0.625 to 2.16 MPa with an average of 1.39 MPa. In Eq. (2), taking ${\sigma }_{\theta }$ = 1.39 MPa, $R$ = 2.5 mm, and $P$ = 100 mmHg, the corresponding thickness was 20 µm approximately. This level of wall thickness can be frequently observed in the thin and transition walls (Fig. 7), suggesting that these walls may undergo a range of wall stress inducing mechanical failures, such as microscopic tears. Since the above utilized the theory of thin-walled pressure vessels, three-dimensional analyses including associated factors such as thickness variations and aneurysmal shapes should be included. Challa and Han (2007) performed nonlinear finite element analyses in aneurysm models. Among their results, the spherical model assuming a uniform thickness of 100 µm gave the wall stress of 0.4 MPa. At $P$ = 100 mmHg, $R$ = 2.5 mm, and *T* = 0.1 mm close to those set by Challa and Han (2007), Eq. (2) gave the wall stress of 0.3 MPa, which was close to their wall stress (0.4 MPa) obtained computationally. These results validated our assessment of wall stress using Eq. (2). In the present study, the aneurysmal wall thicknesses varied by 10–40 times within individual cases, suggesting similar spatial variations of wall stress. In particular, the irregularity of the wall thickness may yield the concentration of wall stress. Challa and Han (2007) reported the thickness variation caused the stress concentration computationally, and the variation of wall thickness and wall stress was related with aneurysmal shapes. Though the above treated aneurysmal walls from mechanical aspects, the alteration of mechanical environments during aneurysmal enlargements may lead to that of biological environments. The emergence of stress concentration may have a potential of inducing SMC apoptosis. If SMC apoptosis is induced mechanically, it would emerge as a cluster due to the nature of stress concentration at a certain point in time and space during aneurysmal enlargement. As reviewed in Introduction, SMC death is an important process in aneurysmal wall pathology (Frösen 2014). SMC apoptosis can be induced by mechanical stretching (Mantella et al. 2015) and has been undeniably found in human intracranial aneurysms, especially in ruptured aneurysms (Frösen et al. 2012). The future study should investigate a linkage between SMC apoptosis and wall mechanics in aneurysmal pathology, which is yet to be investigated so far.

The characteristics of aneurysmal wall thickness suggest a complex interplay of wall injury and repair during aneurysmal enlargement. This interplay may be associated with site-dependent local environments, such as hemodynamics. Aneurysmal enlargement, namely, an enlargement of surface area, is expected to accompany the accumulation of wall stretch. Thin walls can be interpreted as sites exposed to repeated injuries such as microscopic tears inside the wall due to wall stretches without wall repairs upon injuries. Repair of the wall injuries may be related to engraftments of luminal thrombus and the subsequent thrombus organization. Translucent walls are found at locations of flow impingement (Suzuki et al. 2016; Cebral et al. 2019). Flow impingements may inhibit wall repairs hemodynamically by preventing engraftments of luminal thrombus. Thick walls may indicate sites exposed to wall injuries followed by wall repairs. Engraftment of the luminal thrombus may promote SMCs around the injured wall to migrate and proliferate beneath the site under the action of substances released inside the thrombus, such as platelet-derived growth factor. The focal thin and thick spots observed in transition walls can be interpreted as sites exposed to competitive balances between wall injury and repair. Focal thin spots have a chance to be thickened if the wall can be repaired, but the resulting local thickening may yield another stress concentration in the vicinity, which may eventually augment wall stretches and may be followed by the formation of focal thin spots. These competitive balance between wall injury and repair can feasibly induce the formation of focal thin and thick spots.

A trigger of aneurysmal wall injury and repair is still speculative. The modern theory of aneurysmal wall pathology only discusses a pathway of wall injury (Frösen et al. 2012, 2019; Cebral et al. 2017). The counter reaction, namely, a pathway of wall repair, is yet to be explained. Although the modern theory regards hemodynamics as a trigger of wall injury (see Introduction), we alternatively regard blood pressure as a trigger of wall injury. Aneurysmal walls are constantly exposed to wall stress in response to blood pressure. The irregularity of wall thickness may yield stress concentration. If the resulting wall injuries such as microscopic tears are not repaired, these sites may be subject to the accumulation of wall stretch, the reduction of wall thickness, and the inverse augmentation of wall stress. The resulting wall stress has a potential of causing SMC apoptosis (see Introduction). If the SMC apoptosis emerges as a cluster at the site of stress concentration at a certain point, the mechanical strength may be further reduced over time due to the loss of mural cells. The loss of mechanical strength may accelerate the accumulation of wall stretch eventually. Cycling of the interplay between wall mechanics (thickness, stress, stretch, and strength) and cell death (apoptosis) may underly wall thinning and local enlargements of aneurysmal walls.

We propose an alternative theory that blood pressure and hemodynamics have a distinct role in aneurysmal wall remodeling. Blood pressure (wall stress) may act as a trigger of wall injury by inducing wall degenerations through the interplay among wall mechanics and cell death, as explained above. Hemodynamics (impinging flow) may act as an inhibitory factor of wall repair by preventing engraftments of luminal thrombus. The modern theory of aneurysmal wall pathology posits that high wall shear stress triggers endothelial dysfunction, de-endothelialization, and macrophage infiltration. The consequent formation of luminal thrombus induces mural cell death and matrix degradation (Frösen et al. 2012, 2019; Cebral et al. 2017). The theory only focuses on the pathway of wall injuries. The pathway of wall repairs and the possible involvement of blood pressure are neglected. The theory regards hemodynamics and luminal thrombus as a trigger and a promoting factor of wall injury, respectively, which oppose our hypotheses; we regard blood pressure (wall stress) as a trigger of wall injury, while luminal thrombus as a trigger of wall repair and hemodynamics (impinging flow) as an inhibitory factor of wall repair. Our next report would include the verification of our hypotheses in terms of aneurysmal wall ultrastructure using transmission electron micrography.

## Limitations

We begin with the uncertainty estimation of wall-thickness measurements. The shrinkage rate due to tissue dehydration was 30% approximately. We utilized the cadaver-derived intracranial major arteries (ICA and MCA), which had a median wall thickness ranging from 300 to 600 µm in originally prepared conditions (WET). The tissue shrinkage rate may depend on wall thickness. Thicker walls may have a greater shrinkage rate. Since the aneurysmal wall thickness was less than 500 µm, the shrinkage rate of 30% can be regarded as maximum. Therefore, the actual wall thickness of UIAs will likely increase by this proportion at maximum. This proportion may depend on wall thickness, but the value was not accessible. Instead, we adopted the worst-case estimation and assumed a constant regardless of wall thickness. The limitation of spatial resolution may yield measurement uncertainties. The spatial resolution was 5.4–14.6 µm. Taking its average, we assumed ± 5-µm uncertainties. Since we decided the location of innermost and outermost surface for determining the thickness, we regarded the maximum uncertainty of 10 µm. We also set a certain threshold to determine the location of surface. We utilized coated samples, and the contrast was judged to be great enough. Round-off errors in producing polygon models exist but were judged not to amplify the uncertainty of 10 µm. The surface of harvested tissues sometime had adhering substances locally, such as blood-derived coagulates. These can be recognized as they are in three-dimensional STL models and were eliminated as noise. The edge of measured models sometimes included outliers due to the incapability of defining the thickness since the perpendicular line to the middle plane has to contact both the innermost and outermost surfaces simultaneously. We simply excluded edge regions for characterizing the wall thickness in TL and NTL regions and included them in the three-dimensional characterization since the outliers were too small to visible in the figure. From the above mentioned, on top of 30% shrinkage rate as maximum, an uncertainty of 10 µm was assumed regardless of wall thickness and tissue type.

## Conclusion

This study newly characterized the three-dimensional wall thickness of unruptured intracranial aneurysms. Distinct wall profiles revealed three types of aneurysmal wall thicknesses: thin walls, transition walls, and thick walls. The local irregularities of wall thickness may yield the concentration of aneurysmal wall stress in response to blood pressure. The observed thin walls and focal thin spots may be caused by excessive wall stresses at the range of mechanical failure inducing wall injuries, such as microscopic tears. The present results suggested that blood pressure (wall stress) may have a potential of acting as a trigger of aneurysmal wall injury during aneurysmal enlargement.

## Data Availability

The datasets used and/or analyzed during the current study are available from the corresponding author on reasonable request.
